# The Whole Day Movement Matters to Executive Function Among College Students

**DOI:** 10.3390/bs15081129

**Published:** 2025-08-20

**Authors:** Xiaoxia Zhang, Samantha Moss, Caifang Wu, Jean Keller, Xiangli Gu

**Affiliations:** 1 School of Sport Sciences, West Virginia University, Morgantown, WV 26506, USA; 2 Department of Kinesiology, Health Promotion and Recreation, University of North Texas, Denton, TX 76201, USA; samantha.moss@unt.edu (S.M.); jean.keller@unt.edu (J.K.); 3 Department of Kinesiology, University of Texas at Arlington, Arlington, TX 76019, USA; cxw4074@mavs.uta.edu (C.W.); xiangli.gu@uta.edu (X.G.)

**Keywords:** inhibition, movement behavior, screen time, working memory, young adult

## Abstract

“The whole day movement matters to health.” has become an important topic while the associations between executive function and different movement behaviors (i.e., physical activity (PA), sedentary behavior (SB) and sleep) are traditionally examined in isolation. This study aimed to examine the combined associations of movement behaviors (i.e., moderate-to-vigorous PA [MVPA], light PA, screen-based SB, non-screen-based SB, and sleep) with executive function (i.e., working memory, inhibition, and overall executive function) among college students. A total of 366 college students (60.1% female; mean age = 22.59 ± 3.54) were recruited. Validated questionnaires were used to assess light PA, MVPA, screen-based and non-screen-based SB, sleep, and executive function. All the movement behaviors including screen-based SB (*β* = 0.13), sleep (*β* = −0.12), and MVPA (*β* = −0.16) were significant predictors on working memory (R^2^ = 0.09). Only BMI (*β* = 0.14) was found to be a significant contributor to inhibition (R^2^ = 0.05). The overweight/obese group had significantly higher scores (meaning lower functioning) in inhibition compared to peers with healthy weights (d = 0.24). These findings suggest healthy movement behaviors (i.e., engage in MVPA, reduce screen-based SB, sufficient sleep) and weight management are beneficial for executive function during young adulthood.

## 1. Introduction

Executive function (EF) is a set of neurocognitive processes within the prefrontal cortex for purposeful, goal-directed behaviors (Best & Miller, 2010; Carlson et al., 2013). Primarily, executive function has three distinct foundational components, including inhibition, working memory, and cognitive flexibility, which are critical for remembering information, facilitating learning, making decisions, and regulating emotions (Diamond, 2013). Working memory involves aspects of verbal and visuo-spatial functions to recall certain information after completing a task and may have substantial influence surrounding inhibitory response (Baddeley & Logie, 1999; Hester & Garavan, 2005). Executive function develops rapidly during early childhood, continuously through adolescence, and reaches its peak in young adulthood (Best & Miller, 2010; Carlson et al., 2013). However, challenges or deficits in executive function are common among college students and are associated with increased risks and symptoms of anxiety, depression, and Attention-Deficit Hyperactivity Disorder (Jarrett, 2016; Warren et al., 2021) as well as reduced students’ academic performances and problem-solving skills (Southon, 2022). Examining the associations of modifiable behavioral factors with executive function would provide purposeful interventions to address college students’ academic performances and mental wellness.

Physical activity is any bodily movement produced by skeletal muscles that requires energy expenditure, ranging from light intensity tasks like walking to more vigorous activities like sprinting (Caspersen et al., 1985). Regular PA participation, especially at moderate-to-vigorous intensity level, has been shown to enhance working memory, cognitive flexibility, and inhibitory control (Etnier & Chang, 2009; Nakagawa et al., 2020; Salas-Gomez et al., 2020). Despite the benefits of PA, only 46.6% of college students in the U.S. met the recommended PA guidelines (Bailey et al., 2023). Researchers suggest “the whole day matters” (Rollo et al., 2020). This is the concept of 24-h movement behavior that all behaviors in a day, ranging from sleep, sedentary behavior [SB], to vigorous PA interconnect with each other and may synergistically contribute to health-related outcomes (Rollo et al., 2020; Ross et al., 2020; Tremblay et al., 2016). In particular, incorporating various levels of PA intensity (several hours of light PA daily and a minimum of 150 moderate-to-vigorous PA [MVPA] per week), as well as managing appropriate time for SB (i.e., less than 3-h of recreational screen time) and sleep (7–9 h) are recommended to adults for health benefits such as preventing future cognitive decline (Ross et al., 2020; Shalash et al., 2024).

Pedišić’s Activity Balance Model (AB model; Pedišić, 2014) proposes that time spent in different movement behaviors in a day should be balanced to optimal the influence on health outcomes. This model suggests investigating the independent and joint associations of the times spent in each of these movement behaviors with health outcomes. These 24-h movement behaviors will look different for each college student due to their behaviors on campus, creating extremely individualized behavioral patterns. For instance, students who enrolled in later classes experienced longer sleep durations (Onyper et al., 2012), and students who had reported more hours of studying and more credit hours engaged in more SB and less vigorous PA than their counterparts, respectively (Calestine et al., 2017). Understanding and uncovering the complexities of these movement behaviors is warranted in this population.

The need to collectively examine the different types of movement behaviors in a 24-h cycle is needed even though evidence is available of each individual movement behavior’s association with executive function. The existing body of literature among children, adolescents, and older adults have documented that excessive screen-based SB has been linked to diminished executive function (Lissak, 2018; Saunders et al., 2020) while optimal amount of sleep (~7 h) is associated with higher executive function performance, and insufficient or excessive sleep is linked to diminished executive function (Sen & Tai, 2023). Specifically, Phan et al. (2019) suggest a daily sleep of six hours and 37 min for the benefits of increased executive function (i.e., memory capacity) among college students. The few studies that examined two movement behaviors at a time, among young adults, revealed that SB had adverse associations with executive function (i.e., visual attention and task switching) while habitual PA yielded no associations with any of the cognitive tests (Loprinzi & Kane, 2015). In Kato et al. (2018), they studied the combined associations of PA and sleep on cognitive function and concluded that PA was only associated with reaction time, while sleep was only associated with working memory. More research is needed to identify the combined associations of PA, SB, and sleep on executive function for young adults.

Understanding the combined associations among the 24-h movement behaviors is crucial since the positive effects of PA may be diminished by prolonged SB or poor sleep quality (Chaput et al., 2014; Ross et al., 2020). Meanwhile, evaluating the associations between 24-h movement behaviors and executive function has the potential to guide tailored behavioral interventions for college students’ cognition and overall well-being. The purpose of this study was to examine the combined associations of college students’ movement behaviors (i.e., MVPA, light PA, screen-based SB, non-screen-based SB activities, and sleep) with their executive function (i.e., working memory, inhibition, and overall executive function). As a post-hoc analysis, we further assessed the effects of their weight status and sex on movement behaviors and executive function.

## 2. Materials and Methods

A cross-sectional research design was implemented and a total of 366 college students (60.1% female; Mage = 22.59 ± 3.54) were recruited through email at one public university in the southwest region of the U.S., representing various years in college. Half of them were in a healthy weight category (body mass index [BMI] is between 18.5 to 24.9 calculated from a self-reported height and weight). Details of demographic information are presented in Table 1.

The university institutional review board (IRB# 2019-0027) approved the study. Upon IRB approval, a recruitment email and flyer, with the consent form and survey link, were distributed to college students. From the link, students were able to sign the informed consent form prior to starting the online survey. Students who did not consent were automatically withdrawn from the study. Participants were fully informed about the purpose, procedures, risks, and benefits of the research before agreeing to participate and they could refuse and discontinue participation at any time without penalty or loss of benefits. Both the informed consent form and survey were operated through the QuestionPro (Seattle, WA, USA) system.

### 2.1. Instruments

*Executive function*. The Adult Executive Functioning Inventory (ADEXI) was used to assess executive function (Holst & Thorell, 2018). It contains 14 items measuring inhibition (5-item) and working memory (9-item). The sample item for inhibition is “I sometimes have difficulty stopping myself from doing things that I like even though someone tells me that it is not allowed”) and for working memory is “I have difficulties with tasks or activities that involve several steps”). Participants rated themselves on a five-point scale (e.g., 1 = Not True and 5 = Definitely True), with higher scores indicating lower executive function and vice versa. The Cronbach’s alpha in this study was 0.90.

*Physical activity*. The three-item Godin Leisure-Time Exercise Questionnaire (Godin, 2011) was used to assess students’ participation in PA in their leisure time. Participants reported the frequency of participating in vigorous, moderate, and mild/light physical activities, respectively for at least 15 min in their free time in the past seven days. Based on the scoring guideline (Godin, 2011), LPA was calculated as LPA = mild frequency × three metabolic equivalents of task (MET); MVPA was calculated as MVPA = moderate frequency × five MET + strenuous frequency × nine MET, respectively. The calculated index for LPA and MVPA were used in the data analysis.

*Screen-based and non-screen-based sedentary behavior*. Sedentary behavior was measured according to the amount of time engaged in eight sitting behaviors by the Sedentary Behavior Questionnaire for adults (SBQ) (Rosenberg et al., 2010). The SBQ includes screen-based SB (i.e., sitting while watching television, using the computer for study, playing on computer/video games, and talking/texting on the phone) and non-screen based SB (i.e., sitting while listening to music, doing paperwork or office work, reading and doing arts or crafts, and driving/taking car or bus). Each sitting behavior was scaled in nine-time frames per day (i.e., 0, ¼, ½, 1, 2, 3, 4, 5, and above 6 h). The sum of times from the screen-based and non-screen-based behaviors were used in the final data analysis. The Cronbach’s alpha in this study was 0.61.

*Sleep*. Participants’ sleep duration was measured by asking “During the past month, how many hours of actual sleep did you get at each night?” The time in hours and minutes was recorded.

### 2.2. Data Analysis

The data analyses were processed in the SPSS Version 29.0. (Armonk, NY, USA: IBM Corp.). The descriptive statistics including mean and standard deviation were calculated. Pearson product-moment correlation was used to examine the bivariate relationships among study variables. Then three hierarchical linear regressions were processed on inhibition, working memory, and overall executive function, respectively. Because BMI and sex are confounding variables documented to be associated with individual’s movement behaviors (American College Health Association, 2024; Rollo et al., 2020), thus were controlled in the regression models. In each regression model, the step one independent variables were sex and BMI; step two variables were MVPA, light PA, sleep, screen-based SB, and non-screen-based SB. As post-hoc analyses, two multivariate analyses of covariance (MANCOVA) were conducted to examine the effects of weight status (healthy weight vs. overweight/obese) and sex (male vs. female) on executive function and movement behaviors, respectively, after controlling for race and age. An alpha level of 0.05 was used for all data analyses.

## 3. Results

As seen in Table 2, on average, participants slept 6.9 h per day (SD = 1.29) and spent 8.68 h (SD = 3.20) and 4.82 h (SD = 2.86) in screen-based and non-screen-based SB, respectively. The average calculated index for LPA was 11.04 (SD = 7.47) ranging from 3 to 42 and the index for MVPA was 45.58 (SD = 23.91) ranging from 14 to 112. Participants’ average scores of the executive function outcomes were 20.22 (SD = 6.9), 11.58 (SD = 3.59), and 31.81 (SD = 9.57) on working memory, inhibition, and overall executive functioning, respectively. The large standard deviations of executive function outcomes revealed dispersed variations among samples, but ADEXI score has no normative data available thus no cut-off values for consideration of deficits in executive function.

The results of binary relationship analyses were presented in Table 2. Screen-based SB were significantly associated with all three executive function outcomes (rs ranging from 0.12 to 0.16, *p* < 0.05). Non-screen-based SB were significantly associated with overall executive function (r = 0.11, *p* < 0.05) and inhibition (r = 0.13, *p* < 0.05). Sleep (r = −0.12, *p* < 0.05) and MVPA (r = −0.13, *p* < 0.05) had significant associations with working memory, respectively. Sleep was also significantly associated with overall executive function (r = −0.11, *p* < 0.05). BMI was significantly associated with all three executive function outcomes (rs ranging from 0.11 to 0.15, *p* < 0.05).

The results of hierarchical regression models are presented in Table 3. All the movement behaviors including screen-based SB (*β* = 0.13, *p* < 0.05), sleep (*β* = −0.12, *p* < 0.05), and MVPA (*β* = −0.16, *p* < 0.01) were significant predictors on working memory after controlling for other variables and the model explained 9% of the variance (*p* < 0.01). Meanwhile, both screen-based SB (*β* = 0.12, *p* < 0.05) and sleep (*β* = −0.14, *p* < 0.05) were significant behavioral predictors of overall executive function (R^2^ = 0.08, *p* < 0.01) regardless of BMI and sex. In the inhibition model, only BMI (*β* = 0.14, *p* < 0.05) was shown to be a significant contributor to inhibition (R^2^ = 0.05, *p* < 0.05) beyond the contribution from movement behaviors and sex.

Since BMI was shown to be a significant predictor on executive function in the regression analyses and sex is documented as a factor to impact PA participation (American College Health Association, 2024), two MANCOVAs were further conducted to examine the effects of weight status and sex on executive function and movement behaviors, respectively, after controlling for race and age. The results on executive function revealed a significant main effect of weight status (Wilks’ Lambda = 0.98, F_(2, 350)_ = 3.53, *p* < 0.05; η^2^ = 0.02). Specifically, the overweight/obese group had significantly higher scores (meaning lower functioning) in inhibition compared to their peers with healthy weights (M = 12.07 vs. M = 11.20; d = 0.24). There were no main effects of sex and interaction effects observed in executive function outcomes. The results on movement behaviors revealed significant main effects of sex (Wilks’ Lambda = 0.93, F_(5, 331)_ = 5.22, *p* < 0.001; η^2^ = 0.07) and weight status (Wilks’ Lambda = 0.97, F_(5, 331)_ = 2.31, *p* < 0.001; η^2^ = 0.03). Males had significantly higher MVPA (M = 52.38 vs. M = 41.16; d = 0.48) and engaged in significantly less screen-based SB (M = 8.32 vs. M = 9.04; d = 0.24) than females. Compared to the healthy weight group, the overweight/obese group spent significantly more time on screen-based SB (M = 8.56 vs. M = 8.97; d = 0.15). There were no interaction effects of weight status and sex on movement behaviors. Results are presented in Table 4.

## 4. Discussion

The purpose of this study was to examine the combined associations of 24-h movement behaviors with executive function and then to further analyze the effects of weight status and sex on those outcomes among college students. All the movement behaviors including screen-based SB, sleep time, and MVPA, simultaneously and significantly predicted executive function (i.e., working memory) in college students. Screen-based SB and sleep time further predicted overall executive function while BMI significantly predicted inhibition regardless of movement behaviors and sex. These findings highlight the importance of promoting healthy movement behaviors and weight management for better executive function during young adulthood, especially on college campuses. The following paragraphs will proceed with a discussion of all 24-h movement behavior variables’ (MVPA, screen-based SB, and sleep) associations with executive function and will conclude with a deeper exploration into the uncovered weight status and sex disparities among variables.

### 4.1. Associations of 24-h Movement Behaviors with Executive Function

This finding supports the notion that the behaviors in a 24-h cycle are interplaying with each other to synergistically contribute to health-related outcomes such as executive function (Huang et al., 2024; Rollo et al., 2020). It highlights the importance of developing and maintaining healthy movement behaviors including structured PA, planned sleep, and reduction of SB, during young adulthood for better executive function. Contextualizing these movement behaviors with an ecological perspective (Sallis et al., 2006), each movement behavior is impacted through each students’ campus behaviors (i.e., time spent studying, amount of credits).

*Physical activity and executive function.* Our findings continuously supports the benefits of PA for executive function (Álvarez-Bueno et al., 2017; Etnier & Chang, 2009; Felez-Nobrega et al., 2017; Salas-Gomez et al., 2020), it may be of importance to consider the uniqueness of the college students’ campus activities and behaviors when interpreting these findings and for future research targeting these variables in this population. Congruent with the current findings, studies have found that more MVPA substantiate greater working memory benefits than lower-intensity PA (Phan et al., 2019; X. Wang et al., 2023; Yu et al., 2023). Lack of time has been consistently identified as a barrier to engage in PA within college students (Deliens et al., 2015), so performing more vigorous exercise for a shorter amount of time may be a feasible way to support the executive function benefits that PA elicits. It would be also valuable to further study the effects of MVPA dosage on working memory in future research.

*Screen-based behavior and executive function.* Our study found that college students spend over eight (8) hours per day viewing screens, which is in line with previous studies (Nakshine et al., 2022; Roberts et al., 2014). Meanwhile, screen-based SB was significantly and negatively associated with executive function which echoes recent literature (Sarvajna et al., 2024; Tabullo et al., 2024; Tang et al., 2018). Specifically, our findings illustrate that more screen-based SB is associated with lower executive function, such as working memory. Perhaps the seemingly unlimited and constant multi-tasking from various software and devices can create an overwhelming occurrence of stimulation and excessive device reliance to store information rather than using one’s own short-term memory; further, screen-based SB can create distress and deterioration in the prefrontal cortex, which is critical for working memory (Zhou et al., 2019).

*Sleep time and executive function.* Inadequate sleep (<7–9 h per night on a regular basis) may pose a significant issue for optimal physical and cognitive health (Consensus Conference Panel et al., 2015). The current sample reported an average of 6.9 h per night, which is lower than the recommended guideline and their sleep time was favorably associated with executive function. This finding is consistent with previous studies that decreased sleep time may be associated with reduced neural activity in the frontal cortex, which then impairs areas such as working memory (Frenda & Fenn, 2016; Xie et al., 2019).

### 4.2. Weight Status Difference on Executive Function

The current sample revealed about 50% of participants fell into the overweight or obese category according to their BMI. Weight management strategies in this group should be considered since weight status during college years has substantial influence on weight and health status throughout adulthood (Haynos et al., 2018; Votruba et al., 2014). Our findings also revealed that participants in the overweight/obese category experienced worse inhibition than their healthy weight peers. Inhibition relates to the control one can exhibit to maintain their impulse control. It is possible since the overweight/obese group did engage in more screen-based SB, there is a consistent issue in the inability to resist the urge to check their devices/phone which could be the catalyst in the significant differences in executive function that were observed (Pindus et al., 2021). It may be of importance to reduce screentime to enhance college students’ executive function.

### 4.3. Sex Differences on Movement Behaviors

Another important disparity to discuss is related to the significant sex gap (i.e., favoring males) related MVPA participation and screen-based SB. It is vital to note that PA participation in youth will persist into early adulthood years along with a natural decline in engagement upon entering adulthood (Corder et al., 2019). If at an early age, females are not engaging in the recommended amount of MVPA and have excessive SB, it will likely contribute to inactive adulthood lifestyles. Due to substantial benefits of PA on executive function among college students, especially females (Salas-Gomez et al., 2020), it is critical to promote female college students’ PA participation.

### 4.4. Implications

Findings of this study suggest the whole day movement matters to executive function among college students. In the spring of 2025, it was estimated that approximately 18.4 million students were enrolled in postsecondary education institutions as undergraduate and graduate students. Some practical strategies are necessary to develop and maintain healthy movement behaviors on campus. Most colleges and universities have recreational and sports programs where students can increase their physical activity. These physical activity programs need to be accessible, interesting, convenient, and fun. Moreover, such programs may consider providing more structured PA or recreational PA opportunities tailored to female students. Some strategies or initiatives to enhance social support (e.g., friends’ engagement in PA) is also suggested to increase female college students’ MVPA (Young et al., 2018).

Given that college students are notorious for lack of sleep quality/time, obtaining the recommended amount of sleep (7–9 h per night consistently) may not be feasible for all students. Some of the most salient determinants of sleep in college students were identified as well-organized PA and healthy social relations (F. Wang & Bíró, 2021). University wellness programs are encouraged to promote healthy sleeping habits via modifying lifestyle, mental health, and psychosocial and physical factors (F. Wang & Bíró, 2021). To support students’ sleep behaviors, student personnel service professionals may add programming related to teaching calming activities before bedtime, such as meditation, deep breathing, and gentle stretching. It may also be advantageous for college students to nap throughout the day and to limit or eliminate screen-time prior to sleeping to ensure they are receiving adequate rest (Hershner & Chervin, 2014).

Reducing screen-based activities is suggested, such as substituting screentime for time spent outside in nature (Deyo et al., 2024) and substituting screen-based SB with MVPA (Fanning et al., 2017). If substitution of screen time is not possible, other feasible tactics could be implemented such as setting notifications on silent as much as possible, using a passcode instead of Face ID to unlock devices, hiding certain applications on a home screen (Olson et al., 2021), and altering the bright colors of the screen to a grayscale effect (Holte & Ferraro, 2023). Possibly, a course could be added to the college core curriculum where positive lifelong health habits can be explored related to movement behaviors (sleeping, screen time, sedentary behaviors, physical activities) along with other behaviors such as eating, stress, mental well-being.

### 4.5. Limitations and Recommendations

This study contributed to the examination of combined associations of executive function and movement behaviors as this is rarely studied comprehensively in the college student population. This study has limitations. Its lack of generalizability is recognized since this sample is representative of only one university in one region of the United States. Meanwhile, it was a cross-sectional designed study focused on a small sample size of young adults who were at college. The sample may not represent those young adults who did not go to college. The data collected, including the calculation of BMI, was self-reported so assumptions must be made when analyzing and interpreting the data (e.g., answered fully and honestly). Additionally, the ADEXI allows researchers to estimate executive function values, not objectively assess; similarly, the Godin-Shepard Leisure Time scale relies on self-report that only estimation of physical activity values could be used. Although a useful tool, using BMI as a sole measurement of weight status without other anthropometric measures may not adequately capture known health risks (Wu et al., 2024). Future studies should seek to utilize both report-based and laboratory task measures of executive function, as well as both objective and subjective measures of PA, SB, and sleep. Lastly, there is not a normative sample that this study’s data can be compared with, so interpretation of the raw scores for executive function is limited. For future studies of this nature, it’s recommended to broaden the measure of screen-time to include the frequency of checking devices, as this may lead to a more comprehensive investigation towards the improvement of cognitive function (Toh et al., 2021). Future directions may also consider investigating other dimensions of evaluation, such as qualitative assessments, to deepen the understanding of the elements and correlates surrounding executive function. Additionally, inhibition and working memory will decline as an individual ages (as early as 30–40 years old; Ferguson et al., 2021), so future studies may want to consider investigating longitudinal changes from young to middle-adulthood. 

## 5. Conclusions

Movement behaviors in a 24-h cycle interact with each other to jointly contribute to one’s health and well-being. Findings of this study suggest focusing on developing and maintaining healthy movement behaviors like reducing screen-based SB, promoting coping strategies for sleep, and engaging in MVPA throughout the years in school may benefit executive function among young college adults.

## Figures and Tables

**Table 1:** Participants’ Demographic Information.

	Number	%
Sex		
Male	146	39.9
Female	220	60.1
College academic level		
Freshman	25	6.8
Sophomore	29	7.9
Junior	83	22.7
Senior	164	44.8
Unidentified	65	17.8
Race		
Caucasian	113	30.9
Black	59	16.1
Hispanic	85	23.2
Asian	37	10.1
Others	72	19.7
Weight status		
Underweight	5	1.4
Normal weight	190	51.9
Overweight	106	29.0
Obese	65	17.8

**Table 2:** Correlational Analyses Results for Study Variables.

Variable	1	2	3	4	5	6	7	8	9
1. Overall Executive Function	-								
2. Working Memory	0.96 **	-							
3. Inhibition	0.83 **	0.63 **	-						
4. Screen-based SB (h)	0.16 **	0.16 *	0.12 *	-					
5. Non-screen-based SB (h)	0.11 *	0.09	0.13 *	0.17 *	-				
6. Sleep (h)	−0.11 *	−0.12 *	−0.05	−0.04	−0.01	-			
7. MVPA	−0.09	−0.13 *	0.00	−0.07	−0.02	−0.05	-		
8. Light PA	−0.02	−0.03	−0.01	0.02	0.07	0.01	0.36 **	-	
9. BMI	0.13 *	0.11 *	0.15 **	0.11	0.16 **	−0.01	−0.06	−0.06	-
Mean	31.81	20.22	11.58	8.68	4.82	6.89	45.58	11.04	25.55
SD	9.57	6.9	3.59	3.20	2.86	1.29	23.91	7.47	4.98

Note. SB = sedentary behavior; MVPA = moderate-to-vigorous physical activity; PA = physical activity; BMI = body mass index; SD = standard deviation; * means *p* < 0.05; ** means *p* < 0.01.

**Table 3:** Hierarchical Regressions’ Results of Movement Behaviors in Predicting Executive Function Outcomes.

Variable	Working Memory			Inhibition			Overall Executive Function		
	*β*	t	R^2^	*β*	t	R^2^	*β*	t	R^2^
***Step 1***			0.02 *			0.03 *			0.03 *
BMI	0.12	2.07 *		0.16	2.80 *		0.15	2.56 *	
Sex	0.11	1.83		0.01	0.21		0.08	1.41	
***Step 2***			0.09 **			0.05 *			0.08 **
BMI	0.09	1.55		0.14	2.38 *		0.12	2.03 *	
Sex	0.09	1.46		0.02	0.34		0.07	1.18	
Sleep	−0.16	−2.82 *		−0.06	−1.03		−0.14	−2.43 *	
Screen-based SB	0.13	2.20 *		0.10	1.76		0.13	2.26 *	
Non-screen-based SB	0.06	0.99		0.08	1.41		0.07	1.25	
MVPA	−0.16	−2.46 **		0.02	0.37		−0.11	−1.76	
Light PA	0.05	0.76		0.02	0.36		0.04	0.68	

Note. SB = sedentary behavior; MVPA = moderate-to-vigorous physical activity; PA = physical activity; BMI = body mass index; * means *p* < 0.05; ** means *p* < 0.01.

**Table 4:** Weight Status and Sex Differences on Executive Function and Movement Behaviors.

Variables	Male	Female	Healthy Weight	Overweight/Obese
Overall Executive Function	30.95 (10.15)	32.53 (9.17)	31.21 (9.94)	32.62 (9.19)
Working Memory	19.42 (7.11)	20.85 (6.77)	20.02 (7.13)	20.55 (6.72)
Inhibition	11.53 (3.82)	11.67 (3.44)	11.20 (3.52)	12.07 (3.63)
Screen-based SB	8.32 (3.11)	9.05 (2.87)	8.56 (3.26)	8.97 (2.65)
Non-screen-based SB	5.00 (2.80)	4.67 (2.56)	4.58 (2.50)	5.04 (2.81)
Sleep	6.72 (1.31)	7.00 (1.28)	6.80 (1.33)	6.98 (1.26)
MVPA	52.38 (24.12)	41.16 (22.57)	47.37 (25.40)	43.78 (21.91)
Light PA	11.45 (8.46)	10.71 (6.76)	11.35 (7.79)	10.64 (7.14)

Note. SB = sedentary behavior; MVPA = moderate-to-vigorous physical activity; PA = physical activity.

## Data Availability

The data presented in this study are available on request from the corresponding author due to privacy.

## Abbreviations

The following abbreviations are used in this manuscript:

PA	Physical activity
MVPA	Moderate-to-vigorous physical activity
SB	Sedentary behavior
BMI	Body mass index
